# Effect of CD44 Binding Peptide Conjugated to an Engineered Inert Matrix on Maintenance of Breast Cancer Stem Cells and Tumorsphere Formation

**DOI:** 10.1371/journal.pone.0059147

**Published:** 2013-03-18

**Authors:** Xiaoming Yang, Samaneh K. Sarvestani, Seyedsina Moeinzadeh, Xuezhong He, Esmaiel Jabbari

**Affiliations:** 1 Biomimetic Materials and Tissue Engineering Laboratory, Department of Chemical Engineering, University of South Carolina, Columbia, South Carolina, United States of America; 2 Dorn Research Institute, Columbia, South Carolina, United States of America; University of South Alabama, United States of America

## Abstract

**Introduction:**

As cancer cells are affected by many factors in their microenvironment, a major challenge is to isolate the effect of a specific factor on cancer stem cells (CSCs) while keeping other factors unchanged. We have developed a synthetic inert 3D polyethylene glycol diacrylate (PEGDA) gel culture system as a unique tool to study the effect of microenvironmental factors on CSCs response. We have reported that CSCs formed in the inert PEGDA gel by encapsulation of breast cancer cells maintain their stemness within a certain range of gel stiffness. The objective was to investigate the effect of CD44 binding peptide (CD44BP) conjugated to the gel on the maintenance of breast CSCs.

**Methods:**

4T1 or MCF7 breast cancer cells were encapsulated in PEGDA gel with CD44BP conjugation. Control groups included dissolved CD44BP and the gel with mutant CD44BP conjugation. Tumorsphere size and density, and expression of CSC markers were determined after 9 days. For *in vivo*, cell encapsulated gels were inoculated in syngeneic Balb/C mice and tumor formation was determined after 4 weeks. Effect of CD44BP conjugation on breast CSC maintenance was compared with integrin binding RGD peptide (IBP) and fibronectin-derived heparin binding peptide (FHBP).

**Results:**

Conjugation of CD44BP to the gel inhibited breast tumorsphere formation *in vitro* and *in vivo*. The ability of the encapsulated cells to form tumorspheres in the peptide-conjugated gels correlated with the expression of CSC markers. Tumorsphere formation *in vitro* was enhanced by FHBP while it was abolished by IBP.

**Conclusion:**

CD44BP and IBP conjugated to the gel abolished tumorsphere formation by encapsulated 4T1 cells while FHBP enhanced tumorsphere formation compared to cells in the gel without peptide. The PEGDA hydrogel culture system provides a novel tool to investigate the individual effect of factors in the microenvironment on CSC maintenance without interference of other factors.

## Introduction

Breast cancer is the most common type of cancer, which accounts for 23% of all cancers in women worldwide [1]. Breast tumors are highly heterogeneous, in which cells with self-renewal and highly invasive capacity coexist with cells that are more differentiated and non-invasive [2]. Increasing evidence suggests that the heterogeneity of the tumor tissue is rooted in the existence of cancer stem cells (CSCs) [3]. Consistent with this notion, the triple negative breast cancer, which is one of the most aggressive types of breast cancer, contains a high fraction of CSCs [4], [5]. Therefore, understanding the mechanism of CSC maintenance is critical for breast cancer prevention and treatment.

The maintenance of CSCs, like that of normal stem cells, is regulated by the microenvironment. Interactions between the stem cells and support cells, interactions between stem cells and extracellular matrix (ECM), the composition of ECM and the physicochemical properties of the environment are key contributing factors in stem cell maintenance [6]. Two major factors hinder the study of microenvironment on tumor development *in vivo*. First, the process of cancer development takes many years, which makes it difficult to follow. Second, it is difficult to study the effect of a specific factor in the microenvironment while keeping all other factors unchanged, as cancer cells are affected by many factors simultaneously. Many *in vitro* studies have provided insight on the regulation of CSC fate by the microenvironment. However, most *in vitro* studies use 2-dimensional (2D) tissue culture plates coated with ECM components to investigate cell signaling and behavior, which may not reflect those conditions under 3-dimensional (3D) physiological environment. Therefore, the 3D *in vitro* cell culture system has emerged as another approach to investigate the interaction between the microenvironment and cancer cells. Most commonly used matrices for 3D cell culture are type I collagen and Matrigel [7], [8]. However, these matrices contain many cell regulatory factors, which make it difficult to determine the role of individual environmental factors on cell behavior. We have developed an inert polyethylene glycol diacrylate (PEGDA) based *in vitro* 3D cell culture system which does not have cell interaction ligands, thus providing a unique tool to study tumor microenvironment *in vitro*. More importantly, the CSCs of breast cancer can maintain their stemness and proliferate while the growth of non-CSCs is inhibited when encapsulated in the PEGDA gel within a certain range of elastic moduli.

In this study, the inert PEGDA hydrogel, in a certain range of moduli, was used as a 3D cell culture model to investigate the role of cell binding peptides in the maintenance of breast CSCs. More specifically, we investigated the effect of CD44 binding peptide conjugated to the gel or dissolved in the gel on the maintenance of CSCs, because CD44 expression is the most widely used marker for characterization and identification of breast CSCs [9], [10]. CD44 is a cell membrane glycoprotein involved in cell migration and adhesion [11]. CD44 has been used for CSC detection and targeting but the mechanism of its involvement in the maintenance of CSCs is not clear. Antibodies against CD44 inhibit breast tumor growth and prevent cancer recurrence [12]. Anti-CD44 antibodies are found to induce the differentiation of acute myeloid leukemia (AML) stem cells [13]. A CD44 exon v6-specific antibody blocks the metastasis of rat pancreatic cancer cells [14]. Therefore, investigating the role of CD44 binding peptide on regulation of CSCs can provide critical information on the behavior of breast CSCs.

For the purpose of comparison and proof of principle, we also conjugated an integrin binding RGD peptide (IBP) and a fibronectin-derived heparin-binding peptide (FHBP) to the gel to investigate their effects on breast CSC maintenance *in vitro*. We chose those two peptides because fibronectin is one of the major components of ECM that mediates cell adhesion, and integrins are the major receptors on the cell surface that sense the environmental cues. Our results show that conjugation of FHBP to the gel matrix enhanced tumorsphere formation by the encapsulated 4T1 breast cancer cells while CD44BP and IBP abolished sphere formation *in vitro*. Furthermore, results demonstrate that the inert PEGDA hydrogel can be used as a model 3D matrix to study the role of individual factors in the tumor microenvironment on tumorigenesis and maintenance of CSCs.

## Materials and Methods

### Materials

Polyethylene glycol (PEG, nominal molecular weights 4.6 kDa), dichloromethane (DCM), N,N-dimethylformamide (DMF), diisopropylcarbodiimide (DIC), 4-dimethylaminopyridine (DMAP), trifluoroacetic acid (TFA), triisopropylsilane (TIPS), diethyl ether, and hexane were purchased from Acros (Fairfield, OH). The Rink Amide NovaGel™ resin, all Fmoc-protected amino acids, and hydroxybenzotriazole (HOBt) were purchased from Novabiochem (EMD Biosciences, San Diego, CA). Calcium hydride, triethylamine (TEA), paraformaldehyde, 4,6-diamidino-2-phenylindole (DAPI), insulin, penicillin, and streptomycin were purchased from Sigma-Aldrich (St. Louis, MO). Acetomethoxy derivative of calcein (cAM) and ethidium homodimer (EthD) were purchased from Molecular Probes (Life Technologies, Grand Island, NY). Basic fibroblast growth factor (bFGF) and epidermal growth factor (EGF) were purchased from Lonza (Allendale, NJ). Bovine serum albumin (BSA) was obtained from Jackson ImmunoResearch (West Grove, PA). Dulbecco’s phosphate-buffer saline (PBS), trypsin-EDTA, RPMI-160 cell culture medium, fetal bovine serum (FBS), Alexa Fluor® 594 Phalloidin, and Quant-it PicoGreen dsDNA reagent kit were purchased from Invitrogen (Carlsbad, CA). Horse serum and DMEM-F12 medium were purchased from PAA Laboratories (Etobicoke, Ontario) and MediaTech (Manassas, VA), respectively. Spectro/Por dialysis tube (molecular weight cutoff 3.5 kDa) was purchased from Spectrum Laboratories (Rancho Dominquez, CA). DCM was purified by distillation over calcium hydride. All other solvents were reagent grade and were used as received without further purification. The anti-Actin, anti-VEGFa and anti-Vimentin antibodies were purchased from Santa Cruz Biotechnology (Santa Cruz, CA). Fluorescent conjugated secondary antibodies were obtained from Invitrogen. 4T1 mouse breast carcinoma cell line was developed by Dr. Suzanne Ostrand-Rosenberg group and available from ATCC (Manassas, VA) [15]. The cell line was characterized and purified by Dr. Ralph A. Reisfeld at the Scripps Research Institute (La Jolla, CA) [16]. 4T1 cells were a donation from Dr. Reisfeld under a Material Transfer Agreement. MCF7 human breast adenocarcinoma cell line and MCF10a non-tumorigenic epithelial cell line were obtained from ATCC.

### Macromer Synthesis and Characterization

The PEG macromer was functionalized with acrylate groups to produce PEGDA by the reaction of acryloyl chloride with hydroxyl end-groups of PEG. TEA was used as the reaction catalyst. Prior to the reaction, PEG was dried by azeotropic distillation from toluene to remove residual moisture. The polymer was dissolved in dried DCM in a reaction flask and the flask was immersed in an ice bath to cool the solution. In a typical reaction, 5.6 ml acryloyl chloride and 9.7 ml TEA, each dissolved in DCM, were added drop-wise to the reaction with stirring. The reaction was allowed to proceed for 12 h under nitrogen flow. After completion of the reaction, the solvent was removed by rotary evaporation and the residue was dissolved in anhydrous ethyl acetate to precipitate the by-product triethylamine hydrochloride salt. Next, ethyl acetate was removed by vacuum distillation; the macromer was re-dissolved in DCM and precipitated twice in cold ethyl ether. Then, the macromer was dissolved in dimethylsulfoxide (DMSO) and dialyzed against distilled deionized (DI) water to remove the by-products. The PEGDA product was freeze-dried and stored at −20°C. A hydrolytically degradable version of the PEGDA gel (dPEGDA) was synthesized with a similar procedure as described previously [17]. The chemical structure of the functionalized macromer was characterized by a Varian Mercury-300 ^1^H-NMR (Varian, Palo Alto, CA) at ambient conditions with a resolution of 0.17 Hz. The sample was dissolved in deuterated chloroform at a concentration of 5 mg/ml and 1% v/v TMS was used as the internal standard.

### Peptide Synthesis and Characterization

CD44 binding peptide (CD44BP), integrin-binding RGD peptide (IBP), and fibronectin-derived heparin-binding peptide (FHBP) as well as their mutants, selected according to previous reports [18]–[20], were synthesized manually on Rink Amide resin in the solid phase using a previously described procedure [21]. The sequences of these peptides and their mutants are listed in Table 1.

**Table 1:** Peptides and their scrambled sequence.

Peptide name	Sequence	Scrambled (mutant)
CD44BP	RLVSYNGIIFFLK	VLFGFLKIYSRIN
IBP	GRGDS	GRDGS
FHBP	WQPPRARI	RPQIPWAR

Briefly, the Fmoc-protected amino acid (6 eq.), DIC (6.6 eq.), and HOBt (12 eq.) were added to 100 mg resin and swelled in DMF (3 mL). Next, 0.2 mL of 0.05 M DMAP was added to the mixture and the coupling reaction was allowed to proceed for 4–6 h at 30°C with orbital shaking. The resin was tested for the presence of unreacted amines using the Kaiser reagent [21]. If the test was positive, the coupling reaction was repeated. Otherwise, the resin was treated with 20% piperidine in DMF (2×15 min) and the next Fmoc-protected amino acid was coupled using the same procedure. After coupling the last amino acid, the peptides were functionalized with an acrylamide group directly on the peptidyl resin by coupling acrylic acid to the N-terminal amine group under conditions used for the amino acid coupling reaction [21]. The acrylamide-terminated peptide was cleaved from the resin by treating with 95% TFA/2.5% TIPS/2.5% water and precipitated in cold ether. The acrylamide-terminated (Ac) peptides were further purified by preparative HPLC on a 250×10 mm, 10 µm Xterra Prep RP18 column (Waters, Milford, MA) with a flow rate of 2 mL/min using a gradient 5–95% MeCN in 0.1% aqueous TFA at detection wavelength of 214 nm. The HPLC fraction was lyophilized and the product was characterized with a Finnigan 4500 Electro Spray Ionization (ESI) spectrometer (Thermo Electron, Waltham, MA).

### Hydrogel Synthesis and Measurement of Modulus

The PEGDA macromer was crosslinked in aqueous solution to form a gel by ultraviolet (UV) initiated radical polymerization with 4-(2-hydroxyethoxy)phenyl-(2-hydroxy-2-propyl) ketone (Irgacure 2959; CIBA, Tarrytown, NY) photoinitiator. Five mg of initiator was dissolved in 1 mL PBS at 50°C. The macromer was dissolved in PBS by vortexing and heating to 50°C. To prepare 10% PEGDA hydrogel precursor solution, 30 mg PEGDA macromer was mixed with 270 mL of the initiator solution. The hydrogel precursor solution was degassed and transferred to a Teflon mold (5 cm×3 cm×500 µm), covered with a transparent glass plate and fastened with clips. Then, the assembly was irradiated with a BLAK-RAY 100-W mercury long wavelength (365 nm) UV lamp (Model B100-AP; UVP, Upland, CA) for 10 min. Next, disc shape samples were cut from the gel using an 8 mm cork borer and swollen in PBS for 24 h at 37°C. To measure the elastic modulus of the gel, samples were loaded on the Peltier plate of a rheometer (TA Instruments, New Castle, DE) and subjected to uniaxial compressive force at a displacement rate of 7.5 µm/s. The slope of the linear fit to the stress-strain curve for 5–10% strain was taken as the elastic modulus (E) of the gel.

### Cancer Stem Cell Culture and Cell Encapsulation in the Hydrogel

The tumor cells were cultured in RMPI-1640 medium with 10% FBS under 5% CO_2_ at 37°C. Cells were trypsinized after reaching 70% confluency. The synthesized acrylamide-terminated peptides were added to the PEGDA macromer solution and the mixture was sterilized by filtration (220 nm filter). Next, 1.4×10^5^/ml cells (4T1, MCF7, or MCF10a) were added to the macromer solution and mixed gently with a pre-sterilized glass rod. The cell-suspended hydrogel precursor solution was crosslinked with UV for 10 min as described above. After cross-linking, the gel was cut into disks and incubated in stem cell culture medium on ultra-low attachment tissue culture plates under 5% CO_2_. The stem cell medium consisted of DMEM-F12 supplemented with 0.4% BSA, 5 µg/ml insulin, 40 ng/ml bFGF, 20 ng/ml EGF, 5% horse serum, 100 U/ml penicillin, and 100 µg/ml streptomycin [22]. For growing tumorspheres in suspension, trypsinized cells (4T1 or MCF7) were cultured on ultra-low attachment tissue culture plates with stem cell culture medium under 5% CO_2_ at 37°C as described previously [22]–[24]. The gold standard for characterization of CSC tumorspheres for stemness is by the ability to form tumor *in vivo* [25], [26].

### Cell Imaging and Determination of Cell Number

To determine cell viability, gels were stained with cAM and EthD dyes after cell encapsulation to image live and dead cells, respectively. Stained samples were imaged with an inverted fluorescent microscope (Nikon Eclipse Ti-ε, Nikon, Melville, NY). Cell viability was quantified by dividing the image into smaller squares and counting the number of live and dead cells manually. At each time point, three gel samples were removed from the culture medium and stained for imaging. For imaging the encapsulated cells, gels were rinsed twice with PBS and fixed with 4% paraformaldehyde for 3 h. After fixation, cells were permeabilized using PBS containing 0.1% Triton X-100 for 5 min. After rinsing, cells were incubated with Alexa 488 phalloidin (1∶200 dilution) and DAPI (1∶5000 dilution) to stain actin filaments of the cell cytoskeleton and cell nuclei, respectively. Stained samples were imaged with a Nikon Eclipse Ti-ε inverted fluorescent microscope. For determination of cell number, the gel samples were homogenized, cells were lysed, and aliquots were used to measure the double stranded DNA (dsDNA) content using a Quant-it PicoGreen assay as described [27]. Briefly, an aliquot (100 µL) of the working solution was added to 100 µL of the cell lysate and incubated for 4 min at ambient conditions. The fluorescence of the solution was measured with a plate reader (Synergy HT, Bio-Tek, Winooski, VT) at emission and excitation wavelength of 485 and 528 nm, respectively. Measured fluorescent intensities were correlated to cell numbers using a calibration curve constructed with cells of known concentration ranging from zero to 10^5^ cells/mL.

### Real Time PCR Analysis

Total cellular RNA of the gel samples was isolated using TRIzol (Invitrogen) as described [27]. 250 ng of the extracted purified RNA was reverse transcribed to cDNA by SuperScript II Reverse Transcriptase (Invitrogen) with the random primers. The obtained cDNA was subjected to real time quantitative polymerase chain reaction (RT-qPCR) amplification with the appropriate gene specific primers. RT-qPCR was performed to analyze the differential expression of CSC markers CD44, CD24, ABCG2, and SCA1 genes with SYBR green RealMasterMix (Eppendorf, Hamburg, Germany) using Bio-Rad iCycler PCR system (Bio-Rad, Hercules, CA). The expression level of GAPDH gene was used as an internal control. The primers for real time PCR were designed by Primer 3 software (http://frodo.wi.mit.edu). The forward and reverse primer sequences, listed in Table 2, were synthesized by Integrated DNA technologies (Coralville, IA). The relative gene expression levels were quantified by the 2^∧^(–ΔΔC_T_) method as described [28], [29]. Briefly, ΔC_T_ was calculated as ΔC_T_ = C_T_^target gene^−C_T_^GAPDH^. ΔΔC_T_ of the target gene was calculated as ΔΔC_T_ = ΔC_T_^experimental group^−ΔC_T_^reference group^. The reference was the first time point (right after cells were encapsulated in the gel). The relative gene expression (fold-change compared to the reference time point) was calculated as 2^∧^(–ΔΔC_T_).

**Table 2:** Forward and reverse sequence of the PCR primers.

PCR Primer	Forward	Reverse
mouse GAPDH	5′-CATGGCCTTCCGTGTTCC TA-3′	5′-CCTGCTTCACCACCTTCTTGA-3′
mouse CD44	5′-GAA TGTAACCT CCGCTACG-3′	5′-GGAGGTGTTGGACGTGAC-3′;
mouse CD24	5′-CTTCTGGCACTGCTCCTACC-3′	5′-GAGAGAGAGCCAGGAGACCA-3′
mouse ABCG2	5′-AGCAGCAAGGAAAGATCCAA-3′	5′-GGAATACCGAGGCTGATGAA-3′
mouse SCA1	5′-TGGACACTTCTCACACTA-3′	5′-CAGAGCAAGAGGGTCTGCAGGAG-3′
mouse E-Cadherin	5′-ACTGTGAAGGGACGGTCAAC-3′	5′-GGAGCAGCAGGATCAGAATC-3′
mouse N-Cadherin	5′-GGGACAGGAACACTGCAAAT-3′	5′-CGGTTGATGGTCCAGTTTCT-3′
mouse integrin αV	5′-GCTTAAAGGCAGATGGCAAC-3′	5′-AAATGGTGATGGGAGTGAGC-3′
mouse integrin β3	5′-TGACATCGAGCAGGTGAAAG-3′	5′-GAGTAGCAAGGCCAATGAGC-3′
mouse EGFR	5′-CAGTGGGCAACCCTGAGTAT-3′	5′-GGGCCCTTAAATATGCCATT -3′
human GAPDH	5′-GAGTCAACGGATTTG GTCGT-3′	5′-TTGATTTTGGAGGGATCTCG-3′
human CD44	5′-GGCTTTCAATAGCACCTTGC-3′	5′-ACACCCCTGTGTTGTTTGCT-3′
human ABCG2	5′-CACCTTATTGGCCTCAGGAA-3′	5′-CCTGCTTGGAAGGCTCTATG-3′

### Flow Cytometry Analysis

Cells encapsulated in the gel were fixed with 4% paraformaldehyde for 30 min followed by washing with PBS. Next, the gel was incubated in oxidative degradation solution (0.1 M CoCl in 20% hydrogen peroxide) [30]. After the gel was degraded, cells were washed three times with cold PBS containing 5% BSA. MCF7 cells were incubated with phycoerythrin (PE) mouse anti-human CD24 and fluorescein isothiocyanate (FITC) mouse anti-human CD44 (BD Biosciences, Franklin Lakes, NJ), and 4T1 cells were incubated with PE-anti-mouse CD24 and FITC-anti-mouse CD44 (eBioscience, San Diego, CA) in 100 µl PBS with 5% BSA for 45 min on ice in dark. Cells were then washed with cold PBS with 5% BSA three times and analyzed by a flow cytometer (FC500, Beckman Coulter, Brea, CA). Flow cytometry was done multiple times on each sample to ascertain reproducibility of the results.

### Western Blot

The cell encapsulated gel was washed with PBS and homogenized in RIPA buffer (1% NP40, 1% SDS, 150 mM NaCl, 20 mM Tris-Cl pH7.4, 1 mM EDTA protease inhibitors) to extract the proteins. The homogenized sample was centrifuged for 5 min to isolate total proteins. Next, proteins were separated by standard SDS-PAGE using Mini-gel system (Bio-Rad) and transferred to a nitrocellulose membrane by the semi-dry transfer apparatus (Bio-Rad). Membranes were incubated in the blocking buffer (5% fat-free dry milk in TBST buffer) at ambient conditions for 1 h followed by incubation with primary antibodies (1∶200–1∶2000) overnight at 4°C. After washing, the membrane was incubated with HRP-conjugated secondary antibodies for 1 h at ambient conditions. After extensive washing with TBST, the membrane was incubated with ECL detection reagents and exposed to an X-ray film. The intensity of the band was quantified with the Image-J software (National Institutes of Health, Bethesda, MD).

### Tumor Growth *in vivo* and Measurement

To test tumor formation ability of 4T1 cells in the hydrogel, the cell encapsulated gels were cultured *in vitro* in the stem cell medium for 9 days as described above. After tumorsphere formation, gel pieces containing 1×10^5^ tumorsphere cells were implanted subcutaneously in syngeneic Balb/C mice (6 mice/group). Groups included 4T1 tumorsphere cells, grown on ultra-low attachment plates, and injected subcutaneously (control group), degradable version of the PEGDA gel (dPEGDA) without tumor cells (control group), 4T1 cells encapsulated in the dPEGDA and cultured *in vitro* for 9 days prior to implantation, and 4T1 cells encapsulated in CD44BP-conjugated dPEGDA gel and cultured *in vitro* for 9 days prior to implantation. When tumors became measurable, tumor size and growth rate were measured and calculated as described [31], [32]. Mice were euthanized when tumor volume reached above 1000 mm^3^ or 4 weeks after inoculation.

### Ethics Statement

The animal study was carried out according to the guidelines for the care and use of laboratory animals of the NIH and approved protocol by Dorn Research Institute IACUC. Inoculation was performed under isoflurane anesthesia. Animals were monitored daily and all efforts were made to minimize the stress. Since 4T1, MCF7 and MCF10a cell lines were not *de novo* cell lines, ethical committee approval was not required.

### Statistical Analysis

Data were expressed as means±standard deviation. Significant differences between groups were evaluated using a two-way ANOVA with replication test followed by a two-tailed Student’s t-test. To account for multiple pair comparisons, p-values from the t-test were corrected using False Discovery Rate (FDR) method [33]. A value of p<0.05 was considered statistically significant.

## Results

### Sphere Formation in PEGDA Hydrogel

**Figure 1:**
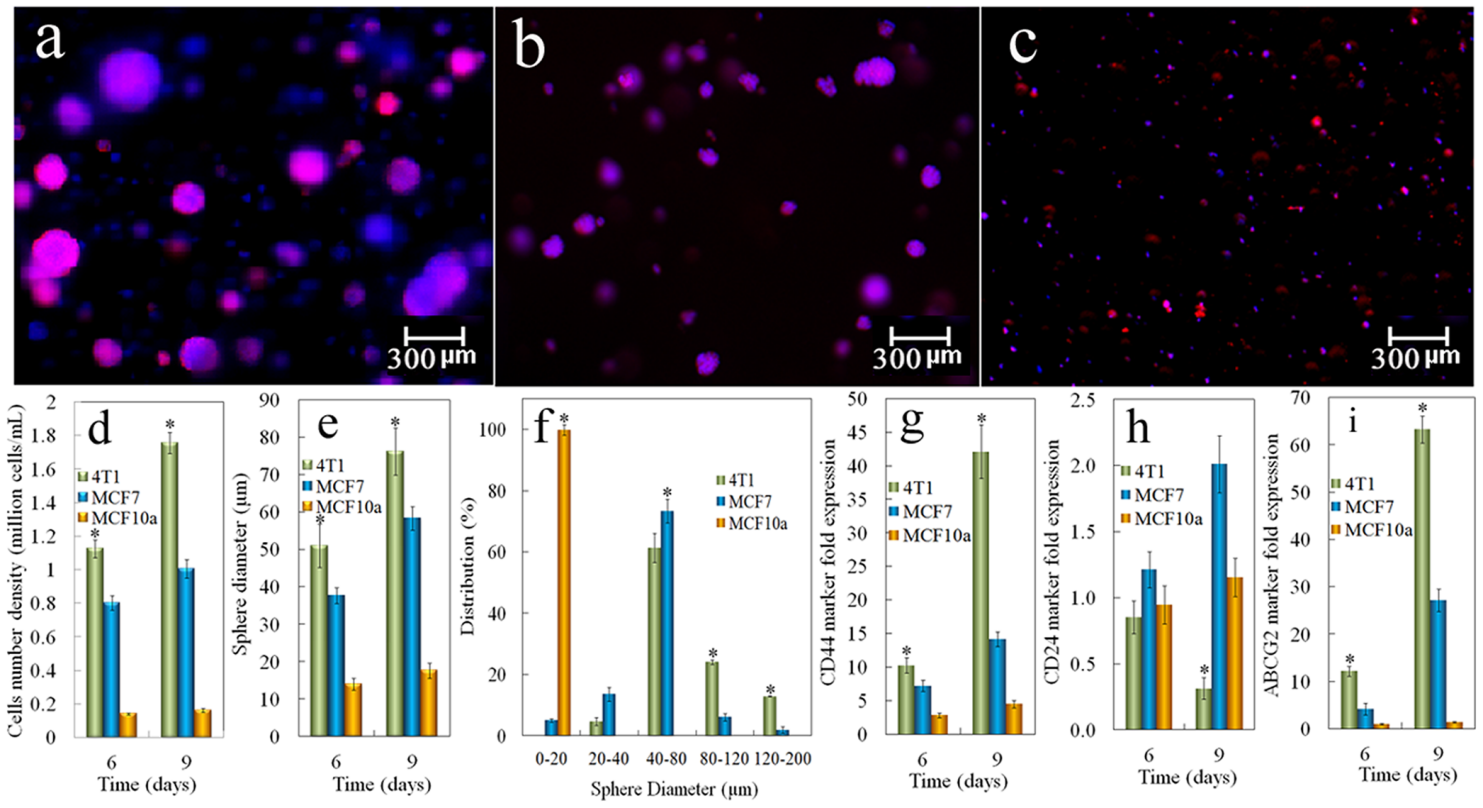
Sphere formation and the effect of cell type encapsulated in PEGDA gel on the expression of CSC markers. Representative fluorescent images of the tumorsphere size and distribution for 4T1 (a), MCF7 (b), and MCF10a (c) cells encapsulated in PEGDA gels (1.4×10^5^ cells/ml), and cultured in stem cell culture medium. Encapsulated cells were stained with phalloidin for cytoskeleton (red) and DAPI for nucleus (blue). Effect of cell type on cell number density (d) and tumorsphere diameter (e) for tumor cells encapsulated in PEGDA hydrogel and incubated in stem cell culture medium for 6 or 9 days. The sphere size distribution (f) was determined 9 days after encapsulation. Effect of cell type on CD44 (g), CD24 (h) and ABCG2 (i) mRNA marker expression for tumor cells encapsulated in PEGDA hydrogel and incubated in stem cell culture medium for 6 or 9 days. RNA levels of the cells were normalized to those at time zero. A star indicates a statistically significant difference (p<0.05) between the test group and the groups with different cell type in the same time point (the same diameter range in f). Values are expressed as mean ± SD (n = 3).

**Figure 2:**
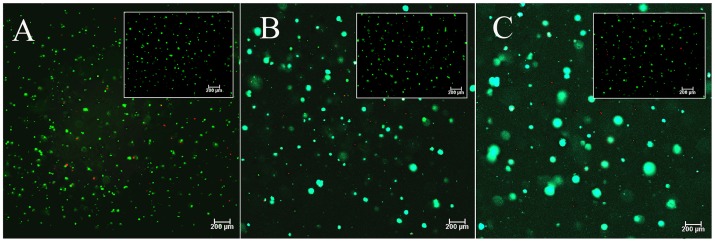
Viability of the cells encapsulated in PEGDA gel. Representative images of live (green) and dead (red) 4T1 cells encapsulated in PEGDA gels with 5 kPa modulus and cultured in stem cell culture medium for 2 (a), 6 (b) and 12 (c) days. Cells were stained with cAM/EthD for live (green) and dead (red) cell imaging. The insets in (a) to (c) are live/dead images of 4T1 cells in PEGDA gels with 70 kPa modulus after 2, 6, and 12 (day), respectively.

We have shown shat tumorsphere formation in the PEGDA gel depended strongly on the gel modulus [34]. Both 4T1 mouse and MCF7 human breast carcinoma cells formed spheres in the gel with 5 kPa modulus, and the sphere formation was correlated with the expression of CSC markers [34]. To determine whether non-cancerous cells could form spheres in the PEGDA gel, MCF10a normal human breast epithelial cells were encapsulated in the gel with 5 kPa modulus and sphere formation was compared with those of 4T1 and MCF7 breast cancer cells. Fluorescent images a–c in Figure 1 show that 4T1 and MCF7 cancer cells encapsulated in the gel formed spheres but not the normal MCF10a cells, suggesting that the spheres originated from the CSC sub-population of 4T1 and MCF7 cancer cells. The cell number density, sphere size, and size distribution for 4T1, MCF7 and MCF10a cells encapsulated in the gel after 6 and 9 days incubation in stem cell culture medium are shown in Figures 1d–1f. The cell density of 4T1 and MCF7 cells significantly increased for both time points, while that of MCF10a remained at a low level (Figure 1d), suggesting that the PEGDA gel promoted the proliferation of tumor cells, but not the normal cells. The density of 4T1 tumorspheres was slightly higher than that of MCF7 after 6 or 9 days of incubation. 4T1 cells also formed larger spheres than MCF7 as shown in Figure 1e. After 9 days of culturing in the gel, nearly 40% of the 4T1 spheres were larger than 80 µm while most of the MCF7 spheres were between 40 and 80 µm (Figure 1f). MCF10a remained as single cells in the gel with size smaller than 20 µm. The expressions of breast CSC markers CD44, CD24, and ABCG2 for the encapsulated cells are shown in Figures 1g–1i. After 6 days of incubation, CD44 expression level in 4T1 and MCF7 cells increased by 10 folds of the initial level (Figure 1g). CD44 expression was further increased in 4T1 cells 9 days after encapsulation. However, as reported previously, the expression of CD44 in 4T1 and MCF7 cells started to decrease after 11 days of incubation irrespective of the extent of cell viability and the increase in tumorsphere size [34]. The expression of CD24 was significantly reduced in 4T1 cells but increased slightly in MCF7 (Figure 1h). Although CD44+/CD24− cells are considered breast CSCs, the expression of CD24 as a CSC marker in MCF7 is not conclusive. Previous studies have indicated that CD44+/CD24− and CD44+/CD24+ cells both display CSC phenotypes in MCF7 cells [35]. The discrepancy may be due to different primers used for real time PCR quantification, and antibodies used for cell sorting as well as the methods used for analysis. The expression of ABCG2, a subunit of ABC transporter that is responsible for the drug resistance of CSCs was also increased in 4T1 and MCF7 cells (Figure 1i). On the other hand, the expression of these markers in MCF10a cells did not change. Figure 2 a to c shows representative images of live (green) and dead (red) 4T1 cells encapsulated in PEGDA gels with 5 kPa modulus after 2 (a), 6 (b) and 12 (c) days, respectively. The insets are the corresponding figures in the 70 kPa gel. For 5 kPa gel, cell viability after 2, 6, and 12 days increased from 91±3% to 94±4% and 97±2%, respectively. For the high modulus 70 kPa gel, no tumorsphere formed and cell viability decreased from 89±4% at day 2 to 84±3% and 78±2% at days 6 and 12, respectively. Based on these results, the effect of peptide conjugation on tumorsphere formation was investigated with 4T1 cells in the PEGDA gel with 5 kPa modulus and incubation time of 9 days.

**Figure 3:**
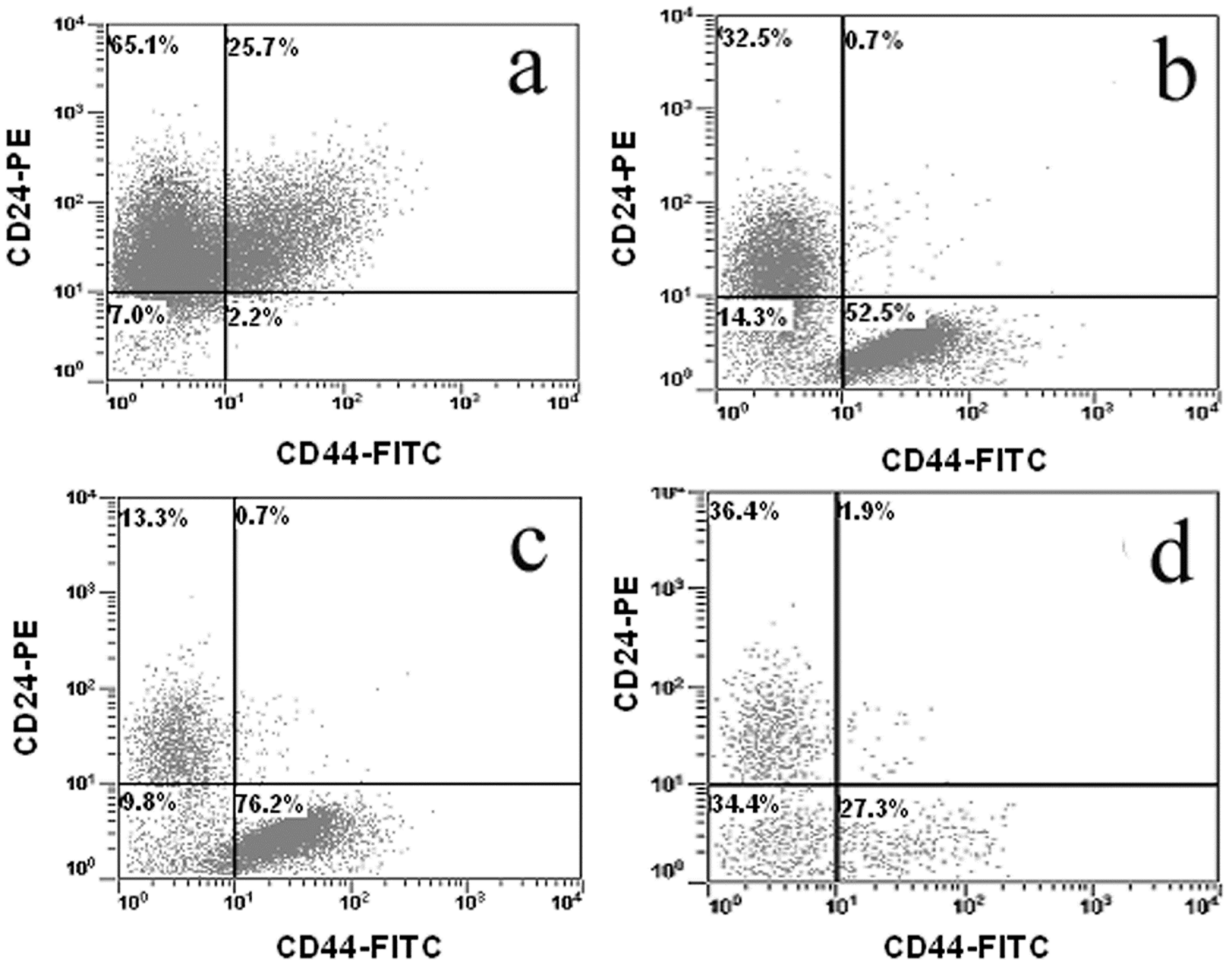
CSC population in the cells encapsulated in PEGDA gel. MCF7 cells were encapsulated in PEGDA gels with 5 kPa modulus and cultured in stem cell culture medium. Cells before encapsulation (a), 3 days (b), 8 days (c) and 11 days (d) after encapsulation were stained with CD44−FITC and CD24-PE antibodies. The population of CD24+, CD44+ and CD44+/CD24− cells was determined by flow cytometry. Flow cytometry was repeated multiple times on each sample to ascertain reproducibility of the results.

The CD44+/CD24− marker expression is widely used for identification of breast CSCs. Flow cytometry analysis of MCF7 cells isolated from the gel is shown in Figure 3. The percentage of CD44+/CD24− cells before encapsulation in the gel was 2% (Figure 3a) but it increased to 53% (Figure 3b) and 76% (Figure 3c) after 3 and 8 days incubation in the gel, respectively. However, the percent CD44+/CD24− cells decreased to 27% after 11 days incubation in the gel (Figure 3d). These results are consistent with our previous results in which the CD44 mRNA expression of 4T1 and MCF7 cells initially increased with time, then began to decrease after 14 days of incubation in the gel [34]. The flow cytometry results demonstrate that the percentage of CSCs in the population of cells encapsulated in the gel increased dramatically after 8 days with incubation time and tumorsphere formation. Since the percentage of live cells in the gel increased with incubation time (see Figure 2), the decrease in the percentage of CSCs at day 11 (Figure 3d) was presumably due to the differentiation of CSCs.

### Effect of CD44 Binding Peptide on Tumorsphere Formation in Hydrogels

**Figure 4:**
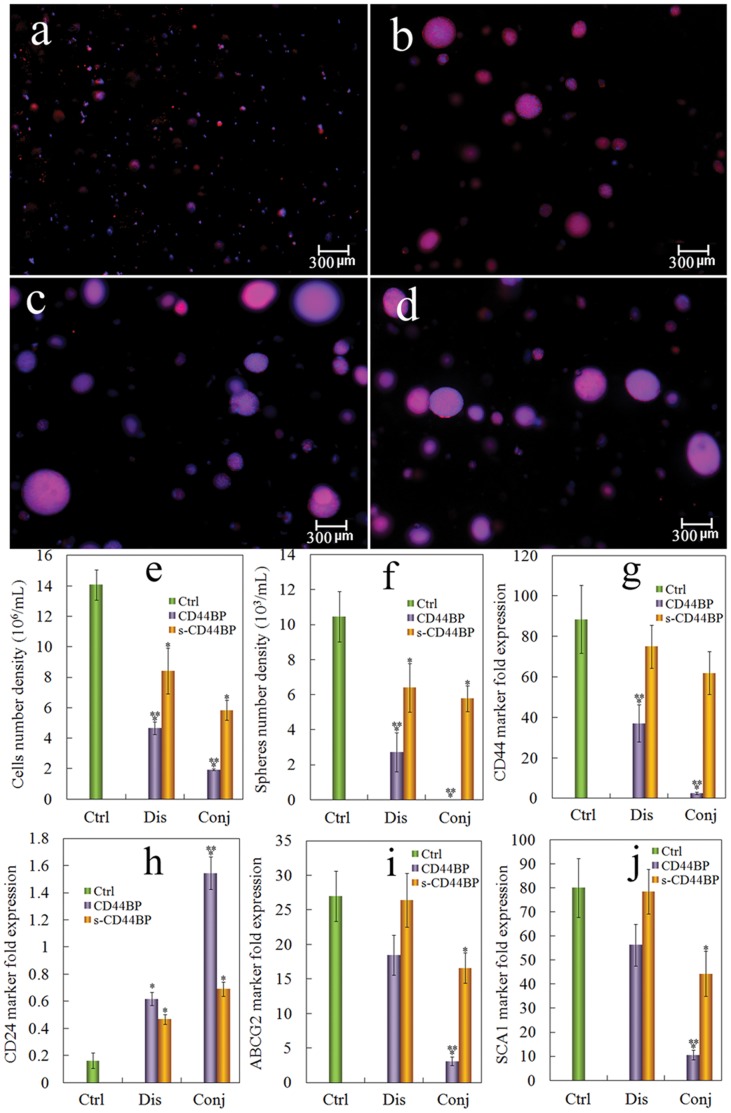
Effect of CD44BP on tumorsphere formation and CSC marker expression. Representative fluorescent images of the tumorsphere size and distribution for 4T1 cells encapsulated in PEGDA gels (1.4×10^5^ cells/ml) conjugated with CD44BP (a, conj CD44BP), conjugated with a scrambled sequence of CD44BP (b, conj s-CD44BP), CD44BP dissolved in the gel (c, dis CD44BP), and s-CD44BP dissolved in the gel (d, dis s-CD44BP) and cultured in the stem cell culture medium for 9 days. Effect of CD44BP on cell number density (e) and tumorsphere number density (f) for 4T1 tumor cells encapsulated in PEGDA hydrogel and incubated in the stem cell culture medium for 9 days. Effect of CD44BP conjugation on CD44 (g), CD24 (h), ABCG2 (i) and SCA1 (j) mRNA marker expression for 4T1 tumor cells encapsulated in PEGDA gel and incubated in the stem cell culture medium for 9 days. RNA levels of the cells were normalized to those at time zero. A star indicates a statistically significant difference (p<0.05) between the test group and “Ctrl”. Two stars indicates a significant difference (p<0.05) between the two CD44BP and s-CD44BP groups within the same form of peptide addition (Dis or Conj). Values are expressed as mean ± SD (n = 3).

It has been shown that CD44BP inhibits breast tumorsphere formation and maintenance of CSCs *in vitro* [18]. The focus of this work was to test the effect of cell binding peptides including CD44BP that interacts with the CD44 receptor up-regulated on CSCs, conjugated to the PEGDA hydrogel on tumorsphere formation in 4T1 tumor cells. Groups included the PEGDA gel without peptide conjugation (control, labeled as Ctrl in Figure 4), the gel with CD44BP or scrambled CD44BP (s-CD44BP) dissolved in the gel and in the culture medium to maintain constant peptide concentration (labeled as Dis), and the gel with CD44BP or s-CD44BP conjugated to the gel (covalent attachment, labeled as Conj). Fluorescent images a–d in Figure 3 show the tumorspheres formed in conj CD44BP, conj s-CD44BP, dis CD44BP, and dis s-CD44BP, respectively. Tumorsphere formation was abolished when 4T1 cells were encapsulated in the CD44BP conjugated gel, indicating the importance of CD44 signaling in the maintenance of CSCs. The effect of CD44BP was consistent with previous reports [18]. However, CD44BP dissolved in the gel (images c and d) did not inhibit sphere formation. These results suggested that CD44BP did not function as a soluble chemokine to inhibit CSC proliferation but functioned within the insoluble part of the ECM. We also tested the effect of a scrambled CD44BP (s-CD44BP). Conjugated or dissolved s-CD44BP had no significant effect on tumorsphere formation, indicating that bioactivity was specific to CD44BP.

Figures 4 e and f show the effect of CD44BP on cell number density and sphere size of 4T1 cells encapsulated in the gel after 9 days of incubation. The 4T1 cell density in the gel reached 14×10^6^/mL after 9 days with 1.4×10^5^/mL initial cell seeding in the gel. The density of 4T1 cells in the gel with CD44BP (conjugated or dissolved and with or without mutation) were lower compared with the gel without any peptide. However, cells in the conj CD44BP gel had the strongest effect on cell density and completely abolished sphere formation (Figure 4a). The expression of CSC markers, CD44, CD24, ABCG2 and SCA1 was also determined and the results are shown in Figures 4 g to j, respectively. 4T1 cells in the CD44BP gel that formed spheres (conj s-CD44BP, dis CD44BP and dis s-CD44BP) had high expressions of CD44, ABCG2 and SCA1 and low expression of CD24. On the other hand, cells in the conj CD44BP gel, which did not form tumorspheres, had decreased expressions of CD44, ABCG2 and SCA1, and increased expression of CD24. These results indicated that tumorsphere formation by 4T1 cells in the gel correlated with the CSC population.

### Effect of CD44 Binding Peptide on Tumor Formation *in vivo*

**Figure 5:**
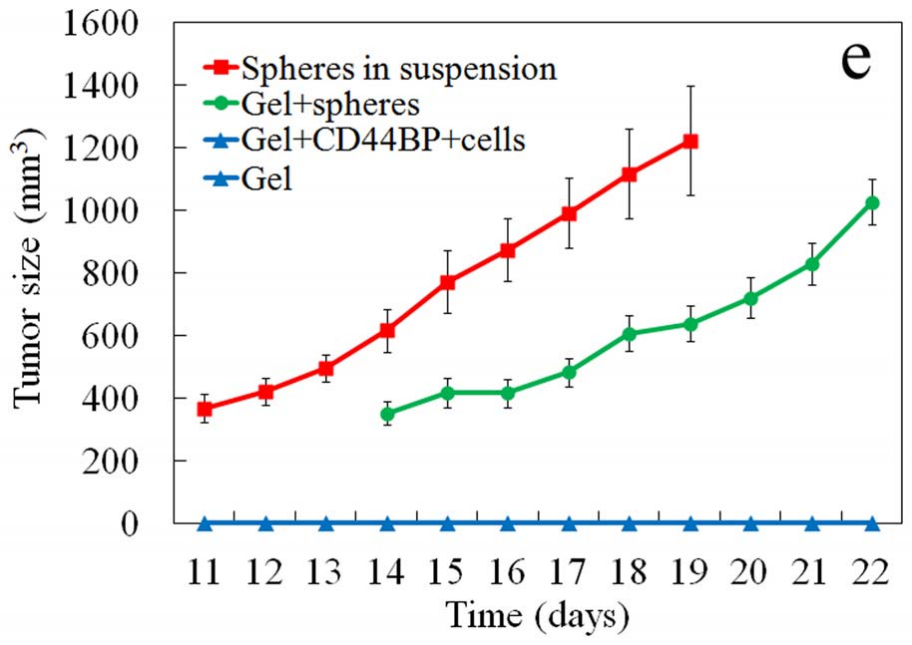
Effect of CD44BP conjugated to the gel on tumor formation *in vivo*. The gel without cell (negative control, light blue), 4T1 tumospheres in suspension (positive control, red), 4T1 cells encapsulated in the gel without CD44BP (green), and 4T1 cells encapsulated in the gel with CD44BP (light blue) were inoculated subcutaneously in syngeneic Balb/C mice. Tumor sizes were measured daily from post-inoculation day 11 (n = 6/group). Tumor growth was not observed in the negative control group (the gel without cell) and the group with 4T1 cells in the gel with CD44BP (the lines for these two groups are overlapped in the figure).

It is well established that tumor growth *in vivo* requires a permissive environment that can support vascularization and matrix remodeling [36], [37]. Therefore, a degradable version of PEGDA gel (dPEGDA) was used to investigate the effect of CD44BP conjugated to the gel on tumor formation *in vivo* by the encapsulated 4T1 cells. Groups included 4T1 tumorspheres injected directly without the gel, gels without cell, gels without peptide conjugation but with 4T1 tumorspheres, and gels with 4T1 cells conjugated with CD44BP. The gels without cell did not from a visible tumor after 4 weeks (Figure 5). Tumors became measurable after 10 days with direct subcutaneous injection of 4T1 tumorspheres. 4T1 tumorspheres in the gel without CD44BP conjugation also formed a tumor after 13 days of inoculation. Even though the formation of tumor was delayed when cells were encapsulated in the gel, the growth rate (the slop of the tumor size curve) did not differ significantly between the group with 4T1 in PBS and 4T1 in the gel (Figure 5). The observed lag time in tumor formation for the encapsulated cancer cells is presumably related to the degradation time of the gel and connection of the tumor cells to the surrounding tissue [17]. However, 4T1 cells encapsulated in the conj CD44BP gel did not form a visible tumor after 4 weeks of inoculation, indicating that CD44BP conjugated to the gel inhibited tumor formation *in vivo*.

### Comparing the Effect of CD44 Binding Peptide on Tumorsphere Formation with Integrin and Heparin Binding Peptides

**Figure 6:**
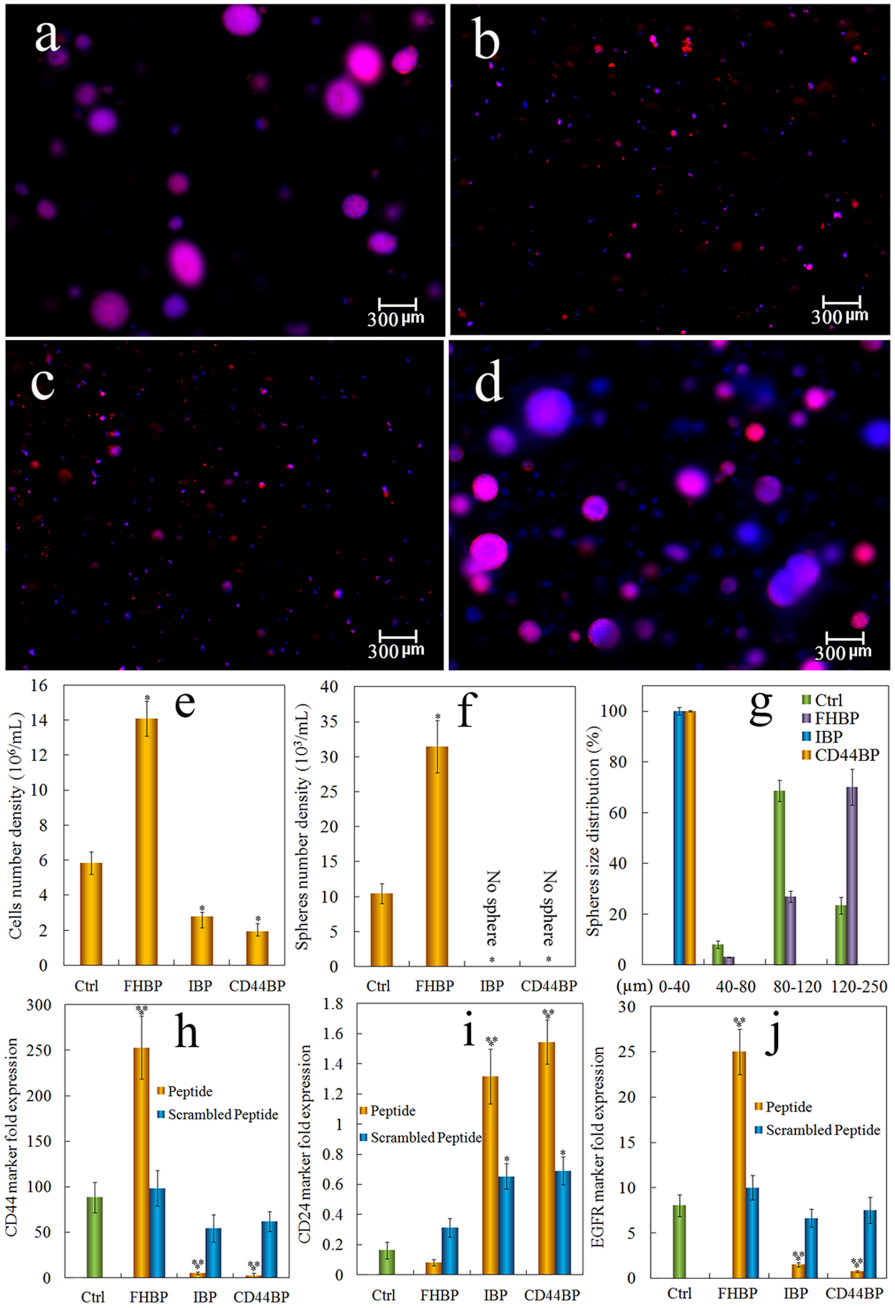
Comparison of tumorsphere formation in PEGDA gels conjugated with CD44BP, IBP, or FHBP. Representative fluorescent images of the tumorsphere size and distribution for 4T1 cells encapsulated in PEGDA gels (1.4×10^5^ cells/ml) without peptide conjugation (a), conjugation with CD44BP (CD44BP, b), conjugation with RGD integrin-binding peptide (IBP, c) and conjugation with fibronectin-derived binding peptide (FHBP, d) and cultured in the stem cell culture medium for 9 days. Effect of cell binding peptide on cell number density (e), tumorsphere number density (f) and sphere size distribution (g) for 4T1 tumor cells encapsulated in PEGDA gel and incubated in the stem cell culture medium for 9 days. Effect of cell binding peptide on CD44 (h), CD24 (i) and EGFR (j) mRNA marker expression for 4T1 tumor cells encapsulated in PEGDA hydrogel and incubated in the stem cell culture medium for 9 days. RNA levels of the cells were normalized to those at time zero. A star indicates a statistically significant difference (p<0.05) between the test group and “Ctrl”. Two stars indicates a significant difference (p<0.05) between the wild type and scrambled peptides for the same conjugated peptide. Values are expressed as mean ± SD (n = 3).

The effect of CD44BP on tumorsphere formation in the gel prompted us to test other cell-binding peptides. IBP, an integrin receptor binding peptide and FHBP, a heparin-binding domain of fibronectin that binds to cell surface heparin sulfate proteoglycans, were conjugated to the gel. Groups included 4T1 cell seeded gel without peptide conjugation, the cell-seeded gel with CD44BP conjugation, the cell-seeded gel with IBP conjugation, and the cell-seeded gel with FHBP conjugation. For determination of marker expression, gels conjugated with a scrambled sequence of the peptides were also tested. Fluorescent images a–d in Figure 6 show sphere formation by 4T1 cells in the gels without peptide, with conj CD44BP, conj IBP, and conj FHBP, respectively. The IBP conjugation, similar to CD44BP, abolished 4T1 tumorsphere formation in the gel (Figure 6c). However, tumorsphere formation increased when 4T1 cells were encapsulated in the FHBP conjugated gel (Figure 6d). Further characterization of the cells in these gels (see Figures 6 e–g) showed that the cells in IBP and CD44BP conjugated gels had reduced cell number, did not form sphere, and remained as single cells or small cell aggregates (<25 µm). On the other hand, the cells in FHBP conjugated gel had higher cell number and larger spheres compared with those in the gels without peptide conjugation.

To determine whether the size and number density of tumorspheres in the gel correlated with the CSC sub-population, CD44 and CD24 expression of the cells in the peptide-conjugated gels were measured. 4T1 cells in the gels without any peptide conjugation and with FHBP conjugation had elevated expression of CD44 marker while the cells in gels conjugated with CD44BP or IBP had decreased CD44 expression (see Figure 6h). More importantly, the CD44 expression in the cells encapsulated in FHBP conjugated gel was significantly higher than that without peptide conjugation. The expression of CD24 in those gels had an opposite pattern to that of CD44 (see Figure 6i). In breast cancer, the expression of epidermal growth factor receptor *(*EGFR) is also closely related to the maintenance of CSCs [38], [39]. The expression of EGFR marker by 4T1 cells encapsulated in the peptide-conjugated gels is shown in Figure 6j. Similar to CD44 marker, the expression of EGFR was increased in the cells encapsulated in FHBP conjugated gel but decreased in CD44BP and IBP conjugated gels. Furthermore, conjugation of a mutant sequence of the peptides to the gel had insignificant or limited effect on tumorsphere formation and the expression of CSC markers, compared to the wild type (see Figures 6 h–j).

**Figure 7:**
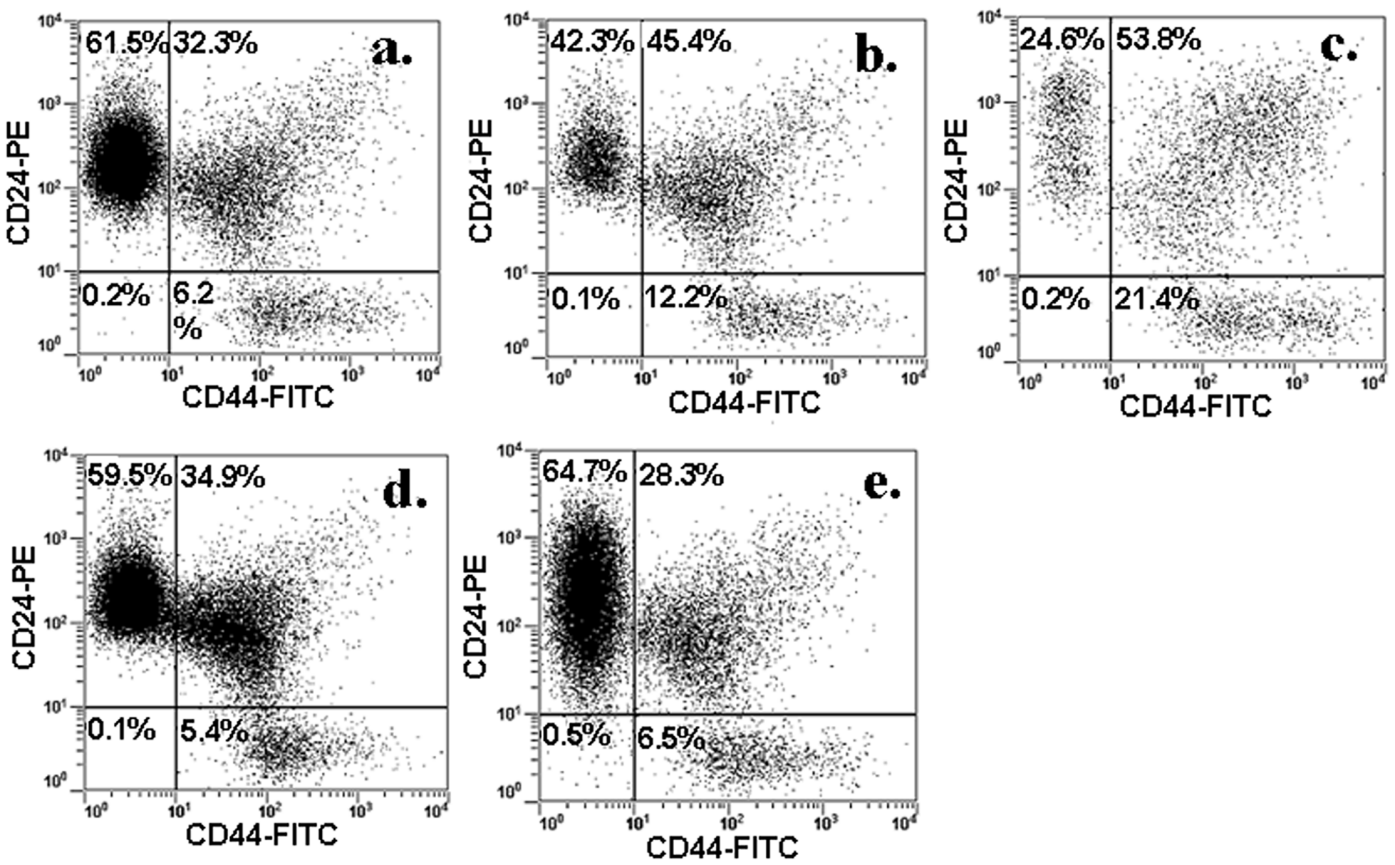
CSC population in cells encapsulated in PEGDA gel conjugated with CD44BP, IBP, or FHBP. 4T1 cells were encapsulated in PEGDA gels with 5 kPa modulus and cultured in stem cell culture medium. Cells before encapsulation (a), 9 days after encapsulation in the gel without peptide (b), 9 days in the gel conjugated with FHBP (c), 9 days in the gel conjugated with IBP (d), and 9 days in the gel conjugated with CD44BP were stained with CD44-FITC and CD24-PE antibodies. The population of CD24+, CD44+ and CD44+/CD24− cells was determined by flow cytometry. Flow cytometry was repeated multiple times on each sample to ascertain reproducibility of the results.

The effect of cell binding peptides on CSC sub-population was further examined in 4T1 cells by flow cytometry. The percentage of CD44+/CD24− cells in 4T1 cells cultured without gel encapsulation was about 6% (Figure 7a). This percentage doubled to 12% for cells encapsulated in the PEGDA gel without peptide conjugation (Figure 7b). When 4T1 cells were encapsulated in the gel conjugated with FHBP, the sub-population of CD44+/CD24− cells was further increased to about 21% (Figure 7c). Conversely, the fraction of CSC sub-population in the gel decreased to the original level (5.4% for IBP and 6.5% for CD44BP) when 4T1 cells were encapsulated in the gels conjugated with IBP (Figure 7d) or CD44BP (Figure 7e) that inhibited sphere formation. These results suggested that tumorsphere formation by 4T1 cells in the gel was related to the CSC sub-population.

### Effects of Cell Binding Peptides on the Expression of Other CSC Related Markers

**Figure 8:**
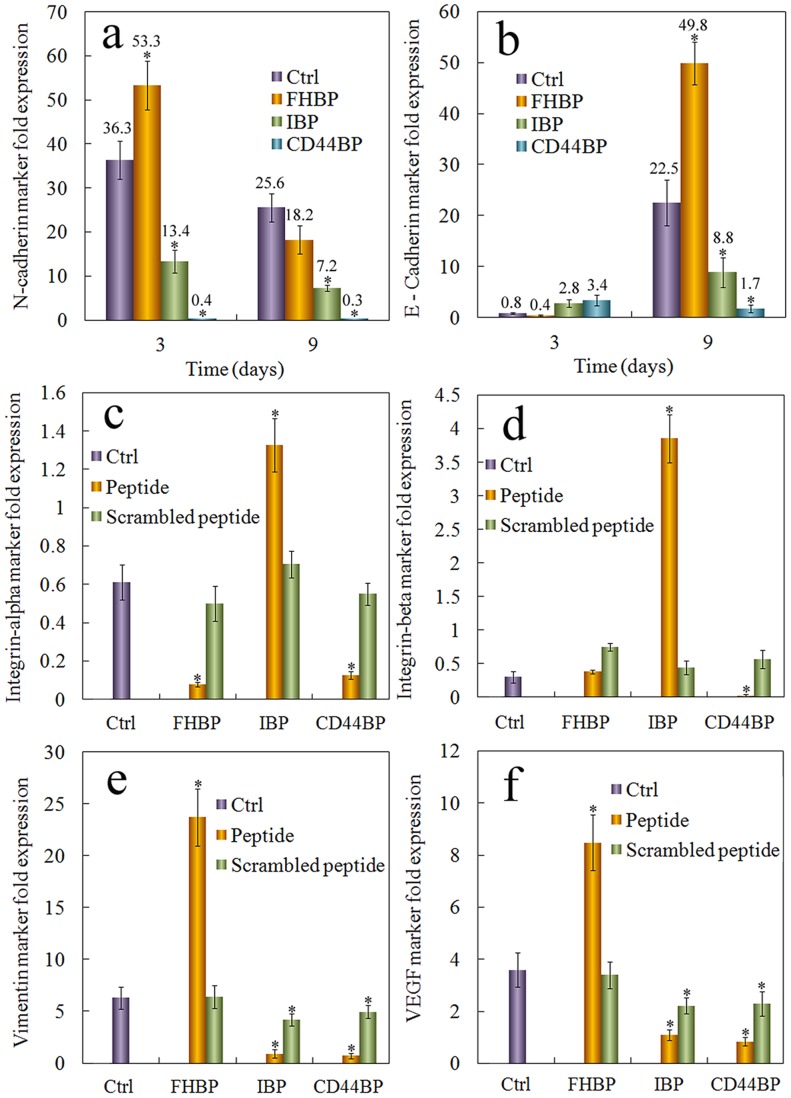
Expression of the markers related to CSC maintenance in cells grown in the gel conjugated with CD44BP, IBP, or FHBP. Effect of cell binding peptide on N-Cadherin (a), E-Cadherin, (b), integrin α_V_ (c), and integrin β_3_ (D) mRNA marker expression for 4T1 tumor cells encapsulated in PEGDA hydrogel and incubated in the stem cell culture medium for 9 days. Effect of cell binding peptide on vimentin (e) and VEGF (f) protein expression. The protein expression was determined by western blot and quantified with imageJ. Actin was used as the internal control and the protein expressions were normalized to those at time zero. RNA levels of the cells were normalized to those at time zero. A star indicates a statistically significant difference (p<0.05) between the test group and “Ctrl”. Values are expressed as mean ± SD (n = 3).

One of the pathways to transform differentiated cancer cells into CSCs is epithelial to mesenchymal transition (EMT) [40], [41]. The hallmark of EMT is the decreased expression of E-Cadherin and increased expression of N-Cadherin [42], [43]. The expressions of E-Cadherin and N-Cadherin 3 and 9 days after cells were encapsulated in the peptide-conjugated gels are shown in Figures 8 a and b, respectively. At the early time point (3 days), the expression of E-Cadherin was decreased while the expression of N-Cadherin was increased in the gel with FHBP, suggesting that EMT was a possible mechanism for the enhanced tumorsphere formation in the gel. However, at the later time point (9 days), the expression of E-Cadherin in the cells grown in FHBP gel was much higher than that in other groups. This was probably due to sphere formation in the FHBP gel. E-Cadherin is a cell adhesion protein and its expression increases with increased cell-cell interaction [44]. Consistent with that, cells in the IBP and CD44BP gels, which did not form spheres, had low expressions of E-Cadherin (see Figure 8b).

The importance of integrins in cancer and CSC maintenance is well known [45]. Since RGD is an integrin binding peptide, we examined the expression of integrin α_V_ and β_3_, two integrin subunits required for RGD binding, and the expressions are shown in Figures 7c and 7d, respectively. The expression of integrin α_V_ and β_3_ was reduced in cells grown in FHBP and CD44BP gels, even though the cells in FHBP gel formed spheres while those in CD44BP gel did not. Interestingly, the expression of integrin α_V_ and β_3_ was significantly increased in the cells in IBP gel. It is possible that blocking integrin signaling by RGD activates a feedback loop to induce integrin expression. These results suggest that the expression of integrin does not correlate directly with tumorsphere formation or CSC maintenance of 4T1 cells. Conjugation of a mutant sequence of the peptides to the gel had limited effect on the expression of CSC markers, indicating that the effect was specific to the wild type.

The expression of vimentin [46] and VEGF, two other markers related to invasive breast cancer and CSC maintenance [47], was also determined at the protein level, as shown in Figure 8 e, f. The expression of vimentin and VEGF was high in cells that formed spheres, and their expression correlated with the sphere size and number (see Figures 6 e–f).

## Discussion

The concept of CSC niche is based on the evidence that both normal stem cells and CSCs utilize similar signaling pathways within a unique microenvironment to maintain stemness. Signaling pathways that have been identified within the stem cell niche include Notch, Hedgehog, PI3K, Wnt, STAT and TGF-β [48]. Processes such as inflammation, EMT, hypoxia and angiogenesis within the microenvironment regulate those pathways to sustain the rare population of CSCs [40].

The cell microenvironment is composed of many cellular and non-cellular components such as cell binding proteins, growth factors, and nutrients. In addition, cells also respond to mechanical properties of the microenvironment such as stiffness and porosity of the ECM [49]. Therefore, cell fate is determined by the specific combination of signals presented in its microenvironment. Due to the complex biochemical composition of the niche, it is difficult to study the role of individual factors in cell behavior with *in vitro* models. We have developed an inert hydrogel as a 3D matrix that supports the proliferation and maintenance of breast CSCs in a certain range of elastic moduli without the interference of proteins, peptides, and other biomolecules in the ECM. In this study, we investigated the effect of conjugating cell-binding peptides to the inert gel with controlled stiffness on the maintenance of stemness of breast CSCs without the interference of other factors.

CD44 is the most widely used marker to identify breast CSCs. CD44 is a cell surface proteoglycan that functions in cell-cell and cell-matrix adhesion. It binds to many ECM ligands including hyaluronic acid (HA), osteopontin, fibronectin and collagen [50]–[53]. It also binds to matrix metalloproteinases (MMPs) and growth factors to promote tumor invasion and growth [54], [55]. Therefore, CD44 utilizes many signaling pathways to regulate cell behavior, and its activity depends on conformational changes and post-translational modifications after ligand binding. CD44BP is a peptide derived from the D-domain of laminin α_5_ chain [18]. It binds to CD44 and inhibits lung colonization of tumor cells *in vivo* but does not inhibit tumor cell proliferation when added to the culture medium [18]. In this study, we found that CD44BP inhibits tumorsphere formation only when conjugated (covalently attached) to the gel. Dissolving the peptide in the gel or in the medium did not have an effect on tumorsphere formation. This result suggests that CD44BP does not act as a soluble chemokine to block or activate CD44 signaling. We speculate that CD44BP induces a conformational change in CD44 receptor through a mechanism like receptor clustering. In previous studies, another CD44 binding peptide, A6, was found to enhance cell adhesion to HA and induce FAK and MEK phosphorylation in a CD44 dependent manner in breast and ovarian cancer cells [56]. A6 also inhibited cancer cell migration and metastasis *in vivo* [56]. It is unclear whether CD44BP binding also causes phosphorylation of these kinases in a CD44 dependent manner.

ECM signaling mainly occurs through the integrin family of proteins [57]. The main integrin receptor is α_V_β_3_ and it has been implicated in the pathogenesis of several types of cancer. Down-regulation of α_V_β_3_ integrin sensitized cancer cells to radiotherapy [58]. Inhibition of α_V_β_3_ integrin blocked the CSC driven tumor formation in a prostate xenograft model [59]. Further studies suggest that blocking α_V_β_3_ integrin leads to redistribution of β-catenin from the nucleus to the cytoplasm [19]. Nuclear localization of β-catenin is known to be one of the mechanisms for maintaining stemness. Therefore, α_V_β_3_ integrin-mediated signaling is required for the maintenance of CSCs. RGD peptide, a well-known ligand of α_V_β_3_ integrin receptor, blocks the integrin mediated cell adhesion. In this study, RGD peptide (IBP) without conjugation to the hydrogel did not affect tumorsphere formation. Since the PEGDA hydrogel did not have cell-binding motifs, blocking cell adhesion sites by RGD might not have a significant effect. However, the matrix rigidity is sensed by the actin cytoskeleton through integrin receptors [60]–[62]. A recent study showed that membrane-bound RGD can induce the clustering of integrin receptors when cells are seeded on RGD peptide conjugated lipid membranes [63]. Furthermore, integrin receptor clustering stimulates local actin polymerization and leads to cytoskeleton remodeling [63]. We speculate that RGD peptide conjugated to the PEGDA hydrogel causes clustering of the integrin receptors, which in turn, alters cells’ ability to sense matrix rigidity. Since tumorsphere formation in PEGDA hydrogel is rigidity dependent, it would be interesting to determine whether breast cancer cells can form spheres in the RGD conjugated PEGDA hydrogel under a different elastic modulus.

The RGD integrin binding peptide is present in many ECM components, but there are other binding motifs in the ECM. For example, fibronectin has a RGD-independent heparin-binding domain in the C-terminus that binds to heparin sulfate proteoglycans on the surface of tumor cells [20], [64]. In this study, we found that unlike CD44 and RGD binding peptides, FHBP conjugated to the gel enhanced tumorsphere formation. It has been shown that FHBP promotes focal adhesion formation in culture cells [20] and it likely activates the focal adhesion kinase (FAK). Several lines of evidence have indicated the role of FAK in promoting breast cancer invasion and metastasis [65], [66] and FAK is required for the survival of breast cancer cells in the absence of cell attachment. The expression of FAK dominant negative mutant in breast cancer cells leads to deactivation and degradation of endogenous FAK and cell apoptosis without matrix attachment [67]. Therefore, FHBP, unlike CD44BP and IBP that block the receptor signaling, may activate FAK and promote CSC survival. As a result, 4T1 cells encapsulated in the gel conjugated with FHBP formed larger and greater number of spheres.

Fibronectin is a mesenchymal marker and its expression is increased during the process of EMT. In the EMT process, certain epithelial cells loose cell-cell adhesion and invade the local tissue. It is thought that a similar transformation occurs during cancer metastasis [68]. Recent studies have shown that EMT induction is sufficient to turn differentiated cancer cells into CSCs [40], [41]. A recent study showed that mammary tumor cells displayed a more differentiated phenotype when cultured on collagen coated substrates, while they displayed an invasive phenotype and EMT-related gene expression pattern when cultured on fibronectin coated substrates [69]. Therefore, FHBP may induce EMT which in turn enhance tumorsphere formation. In the EMT process, the expression of E-Cadherin is down regulated but E-Cadherin may also be up-regulated with increasing cell-cell interaction. Consistent with this, the expression of E-Cadherin was initially reduced in our study in the cells encapsulated in FHBP gel but increased at a later time point. The expression pattern of some of the examined EMT markers also suggests that enhanced EMT may contribute to the increased tumorsphere formation in FHBP conjugated gels.

This study also included the mutant forms of the peptides. Although the mutants did not affect tumorsphere formation, they slightly affected the expression of some of the markers. It is possible that the mutants bind to the corresponding receptors but with much lower affinity or non-specifically. In summary, this study demonstrated that cell adhesion peptides could either increase or diminish CSC population in the inert 3D PEGDA hydrogel cell culture system, as the mechanisms for CSC maintenance among these peptides are different.

### Conclusion

Using the 3D PEGDA matrix with a certain stiffness, we demonstrated that the cell adhesion CD44 binding peptide (CD44BP), RGD integrin binding peptide (IBP), and fibronectin-derived heparin binding peptide (FHBP) can be individually conjugated to the inert PEGDA gel and their effect on the maintenance of breast cancer stem cells can be investigated without the interference of other factors. The CD44BP and IBP conjugated to the inert gel completely abolished tumorsphere formation by the encapsulated 4T1 breast cancer cells while FHBP enhanced tumorsphere formation compared to those without peptide. The inert 3D hydrogel cell culture system provides a novel tool to investigate the individual effect of factors in the microenvironment on maintenance of CSCs without the interference of other factors.
